# Clinicopathological characteristics of adrenocortical carcinoma in the Kyushu–Okinawa area of Japan

**DOI:** 10.1111/iju.15386

**Published:** 2024-01-09

**Authors:** Shotaro Nakanishi, Yumi Fukushima, Junichi Inokuchi, Tomoaki Hakariya, Hiroaki Kakinoki, Hideki Enokida, Katsuaki Chikui, Hirofumi Matsuoka, Toshitaka Shin, Shoichiro Mukai, Tomomi Kamba, Masatoshi Eto, Ryoichi Imamura, Mitsuru Noguchi, Tsukasa Igawa, Nobuhiro Haga, Toshiyuki Kamoto, Naohiro Fujimoto, Seiichi Saito

**Affiliations:** ^1^ Department of Urology, Graduate School of Medicine University of the Ryukyus Nishihara Japan; ^2^ Department of Urology, Faculty of Life Sciences Kumamoto University Kumamoto Japan; ^3^ Department of Urology, Graduate School of Medical Sciences Kyushu University Fukuoka Japan; ^4^ Department of Urology Nagasaki University Graduate School of Biomedical Sciences Nagasaki Japan; ^5^ Department of Urology, Faculty of Medicine Saga University Saga Japan; ^6^ Department of Urology, Faculty of Medicine Kagoshima University Kagoshima Japan; ^7^ Department of Urology Kurume University School of Medicine Kurume Japan; ^8^ Department of Urology, Faculty of Medicine Fukuoka University Fukuoka Japan; ^9^ Department of Urology, Faculty of Medicine Oita University Yufu‐Shi Japan; ^10^ Department of Urology, Faculty of Medicine University of Miyazaki Miyazaki Japan; ^11^ Department of Urology, School of Medicine University of Occupational and Environmental Health Kitakyushu Japan

**Keywords:** adjuvant treatment, adrenocortical carcinoma, surgical role

## Abstract

**Objective:**

Adrenocortical carcinoma is a rare condition, with limited comprehensive reports from Japan. This study aimed to review Japan's data on adrenocortical carcinoma by assessing information from 46 patients—with adrenocortical carcinoma across 10 Japanese university hospitals.

**Methods:**

We conducted a retrospective multi‐institutional analysis of the clinical characteristics of adrenocortical carcinoma in Japan. We evaluated data from 46 patients across 10 university hospitals over 10 years and analyzed the relationship between clinicopathological characteristics and overall survival.

**Results:**

Five‐ and 10‐year overall survival rates were 59% and 53%, respectively. Overall survival was significantly different among the tumor–node–metastasis system for adrenocortical carcinoma of the American Joint Committee on Cancer/International Union Against Cancer, with the worst prognosis in stage IV (*p* = 0.0044). In our cohort, neither the Weiss score nor the Ki‐67 proliferation index correlated with overall survival. Adjuvant treatment did not yield improved overall survival, whereas resection of the primary tumor in stage IV disease was significantly associated with improved overall survival (*p* = 0.0262). Out of the cases evaluated for plasma hormones, plasma cortisol, aldosterone, testosterone, and DHEA‐S levels were measured at 23%, 42%, 29%, and 62%, respectively, demonstrating higher levels than the upper normal limits.

**Conclusion:**

Patients with stage IV adrenocortical carcinoma had a poor prognosis; however, resection of the primary tumor in stage IV disease was associated with prolonged survival. The results of this study are expected to contribute to future treatment of adrenocortical carcinoma in Japan.

**Abbreviations & Acronyms**
ACC: adrenocortical carcinoma
DHEA‐S: dehydroepiandrosterone‐sulfate
ECOG PS: Eastern Cooperative Oncology Group Performance Status Scale
ENSAT: European Network for the Study of Adrenal Tumors
TNM: tumor–node–metastasis

## INTRODUCTION

Adrenocortical carcinoma (ACC) is a rare disease, with an annual incidence of approximately 0.7–2 cases per million people.^1^ The prevalence rate is higher in women aged 40–50 years. Most cases of ACC are sporadic, although there are some congenital and familial forms of the disease.^2^ ACC is suspected when the tumor size exceeds 4 cm, because ACC tumors are larger than benign adrenal masses. ACC accounts for 2% of adrenal tumors measuring 4 cm or less, 6% of tumors that measure from 4.1 to 6 cm, and 25% of tumors greater >6 cm.^3^ Surgical resection is the mainstay of treatment for the localized disease.^4^ Although most patients with ACC present with a resectable disease, the recurrence rate is high. Therefore, adjuvant mitotane therapy has been reported to significantly prolong recurrence‐free survival in a study using multivariate analysis.^5^ Although patients with European Network for the Study of Adrenal Tumors (ENSAT) stage IV have poor prognosis, cytoreductive adrenalectomy significantly improves overall survival.^6^ However, due to the limited treatment methods available at present, a novel approach for ACC is urgently required.

Because ACC is derived from endocrine organs, hormone‐secreting tumors account for approximately 60% of all ACC cases. However, symptoms caused by hormone over‐secretion are present in approximately 40% of all cases of ACC.^7^ Patients with hormone‐secreting ACC present with manifestations of virilization, feminization, Cushing syndrome, or rarely hyperaldosteronism, whereas nonfunctioning ACC is usually diagnosed incidentally.

Despite being a rare cancer, comprehensive reports on ACC from Japan are limited. Therefore, it is essential to review Japan's data on ACC. In this study, we aim to assess data on 46 patients—with ACCs—across 10 Japanese university hospitals.

## METHODS

We retrospectively analyzed the medical records of patients with ACC, who underwent treatment across 10 university hospitals in the Kyushu and Okinawa districts between January 2009 and May 2019. This study was approved by the Ethics Committee of the University of the Ryukyus and each Institution (Approval No. 1442) and each hospital.

The diagnosis and therapeutic care of the ACC patients were based on the General Rule for Clinical and Pathological Studies on Adrenal Tumor^8^ and the guidelines of the ENSAT group.^4^ The staging system was based on the tumor–node–metastasis (TNM) system for ACC of the American Joint Committee on Cancer/International Union Against Cancer, which has been available since 2009.^9^ Stage I was defined by the presence of a tumor with a diameter of <5 cm (T1N0M0); stage II was defined by the presence of a tumor with a diameter of >5 cm (T2N0M0); stage III was defined by any size with local invasion but not invading adjacent organs, or tumor greater than 5 cm in size and regional lymph node metastasis (T1‐2N1M0 or T3N0M0); stage IV was defined by the presence of distant metastasis or any size that invades adjacent organs or large blood vessels (T4N0M0 or T3‐4N1M0 or any T any N M1). Recurrence of disease was based on clinical, laboratory, and radiological evidence and did not require histological confirmation.

Plasma hormone hypersecretion should be suspected in adrenal tumors, especially when a patient presents with clinical symptoms. The reference values for the adrenocortical hormone levels were as follows: adrenocorticotropic hormone: 9–52 pg/mL; testosterone: male, 2.0–7.5 ng/mL and female, 0.1–0.5 ng/mL; cortisol: 2.7–15.5 μg/dL; aldosterone: 30–160 pg/mL; dehydroepiandrosterone‐sulfate (DHEA‐S): male, age range: 20–29; 165–542 μg/dL, 30–39; 120–441 μg/dL, 40–49; 83–396 μg/dL, 50–59; 62–282 μg/dL, more than 60; 14–224 μg/dL, female, age range: 20–29; 85–299 μg/dL, 30–39; 54–203 μg/dL, 40–49; 25–195 μg/dL, 50–59; 11–116 μg/dL, more than 60; 50–100 μg/dL. Therefore, we defined an abnormal level of hormone as follows: adrenocorticotropic hormone ≤5 pg/mL; testosterone ≥10 ng/mL; cortisol ≥20 μg/dL; aldosterone ≥150 pg/mL; and DHEA‐S: It was defined as being above the upper limit of normal for age and gender.^10^, ^11^, ^12^

Statistical analyses were performed using JMP version 15 (SAS Institute Inc., Cary, NC, USA). Survival time was calculated from the time of diagnosis to the date of death. The relationship between categorical variables and overall survival was analyzed using the Kaplan–Meier method, and statistical significance was calculated using the log‐rank test. Statistical significance was set at *p* < 0.05.

## RESULTS

### Backgrounds of the patients

We enrolled 46 patients with ACC (Table 1). The diagnosis of ACC was based on resected specimens of adrenalectomy in 34 cases and metastasectomy in two cases, while the remaining 10 cases were diagnosed by needle biopsy (one case), autopsy (two cases), and imaging testing (seven cases). We presented details of seven cases diagnosed with imaging. The median size of the primary tumor in these cases was 75 mm (50–105). Among them, three cases underwent fluorodeoxyglucose positron emission tomography, which showed high uptake, while five cases were diagnosed by computed tomography and magnetic resonance imaging.

**TABLE 1:** Clinicopathological background of the ACC patients.

	No. or median	% or range
Age (years): median, range	60.5	(16–83)
Men/Women	16/30	35%/65%
BMI, median, range	22.5	(14.8–38)
PS		
0	27	59%
1	12	26%
2	1	2%
Unknown	6	13%
Tumor laterality		
Right	14	30%
Left	28	61%
Unknown	4	9%
Tumor size, median, range (mm)	73.5	(11–200)
Comorbidities		
Diabetes	8	17%
Hypertension	24	52%
Hyperlipidemia	8	17%
Opportunity for discovery		
Symptomatic	20	43%
Asymptomatic	25	54%
Unknown	1	3%
Diagnostic methods		
Adrenalectomy	34	74%
Metastasectomy	2	4%
Needle biopsy	1	3%
Autopsy	2	4%
Imaging test	7	15%
Clinical T stage		
1	6	13%
2	22	48%
3	7	15%
4	9	20%
Unknown	2	4%
N stage		
0	32	70%
1	10	21%
Unknown	4	9%
M stage		
0	31	67%
1	13	28%
Unknown	2	5%
Metastatic sites		
Lung	6	13%
Liver	5	11%
Lymph nodes	2	4%
Bone	1	2%
Others	6	13%
AJCC 7th staging		
I	3	7%
II	19	41%
III	8	17%
IV	14	30%
Unknown	2	5%
Weiss criteria (*N* = 28)		
3	5	18%
4	8	29%
5	5	18%
6	3	11%
7	4	14%
8	2	7%
9	1	3%
Ki‐67 index (*N* = 26)		
More than 10%	14	54%
Pathological T stage (*N* = 34)		
1	3	9%
2	12	35%
3	5	15%
4	1	3%
Unknown	13	38%

Of the 34 patients, six were diagnosed with benign tumors and underwent surgery. Fifteen patients underwent laparoscopic surgery, with 12 having confirmed negative surgical margins and three with unknown margin. On the other hand, 18 patients underwent open surgery. Surgical margins were negative in 10 cases, positive in two cases, and unknown in the remaining six cases. The median tumor diameter in cases treated with laparoscopic surgery was 52 mm, while it was 82.5 mm in cases treated with open surgery (*p* = 0.0015) (data not shown).

The opportunities for diagnosis included clinical symptoms in 20 patients (43%) and incidental in 25 (54%). Thirty (65%) patients were female. The median age at diagnosis was 60.5 years. Approximately 85% of the patients had an Eastern Cooperative Oncology Group Performance Status Scale (ECOG PS) <2. Among the comorbidities, hypertension was the most common in 24 patients (52%). The tumor staging was as follows: 3 (7%) stage I, 19 (42%) stage II, 7 (15%) stage III, and 14 (29%) stage IV. The most common site of metastasis was the lungs in six cases (13%), followed by liver in five cases (11%). The Weiss score was calculated for 28 tumor sections. Weiss scores 3–5 corresponded to 65% of the cases (Table 1). The Ki‐67 proliferation index (PI) was examined in 26 patients, of whom, 14 patients (54%) had more than 10% of the PI.

### Treatment for the ACC patients

Thirty‐four patients (74%) underwent adrenalectomy. Of these, 18 patients (53%) underwent open surgery and 15 (44%) underwent laparoscopic surgery. The procedures involved adrenalectomy alone in 26 patients (76%), adrenalectomy with dissection of regional lymph nodes in three patients (9%), and adrenalectomy with combined resection of the surrounding organs and metastasectomy in five patients (15%). The median operating time was 230 min (IQR: 206.5–319), and the blood loss was 320 mL (29–1455).

Only one patient (3%) received neoadjuvant chemotherapy, which consisted of mitotane plus etoposide, doxorubicin, and cisplatin (EDP). EDP is a chemotherapy that combines etoposide, doxorubicin, and cisplatin. The doses and administration of these medications were as follows: etoposide: 100 mg per square meter of body‐surface area on days 2 to 4, doxorubicin: 40 mg per square meter on Day 1, and cisplatin: 40 mg per square meter on Days 3 and 4. Adjuvant chemotherapy was administered to 19 patients (59%). Seventeen patients received mitotane, one received mitotane plus EDP, and the other had incomplete information (Table 2). Of 19 patients who had adjuvant therapy, Ki‐67 PI was examined in 12; half of these patients were classified as high risk (Ki‐67 PI exceeding 10%), while the other half were categorized as low risk.

**TABLE 2:** Treatment for the ACC patients.

Surgical methods (adrenalectomy *n* = 34)		
Open surgery	18	53%
Laparoscopic surgery	15	44%
Unknown	1	3%
Resection		
Adrenalectomy alone	26	76%
Added lymph node resection	3	9%
Added surrounding organ resection and metastasectomy	5	15%
Operative time	230	[206.5–319]
Blood loss	320	[29–1455]
Neoadjuvant chemotherapy		
Received	1	3%
Mitotane plus EDP	1	
Not received	28	87.5%
Unknown	3	9.5%
Adjuvant chemotherapy		
Received	19	59%
Mitotane only	17	
Mitotane plus EDP	1	
Unknown	1	
Not received	13	41%

### Outcome of the patients with ACC

**FIGURE 1:**
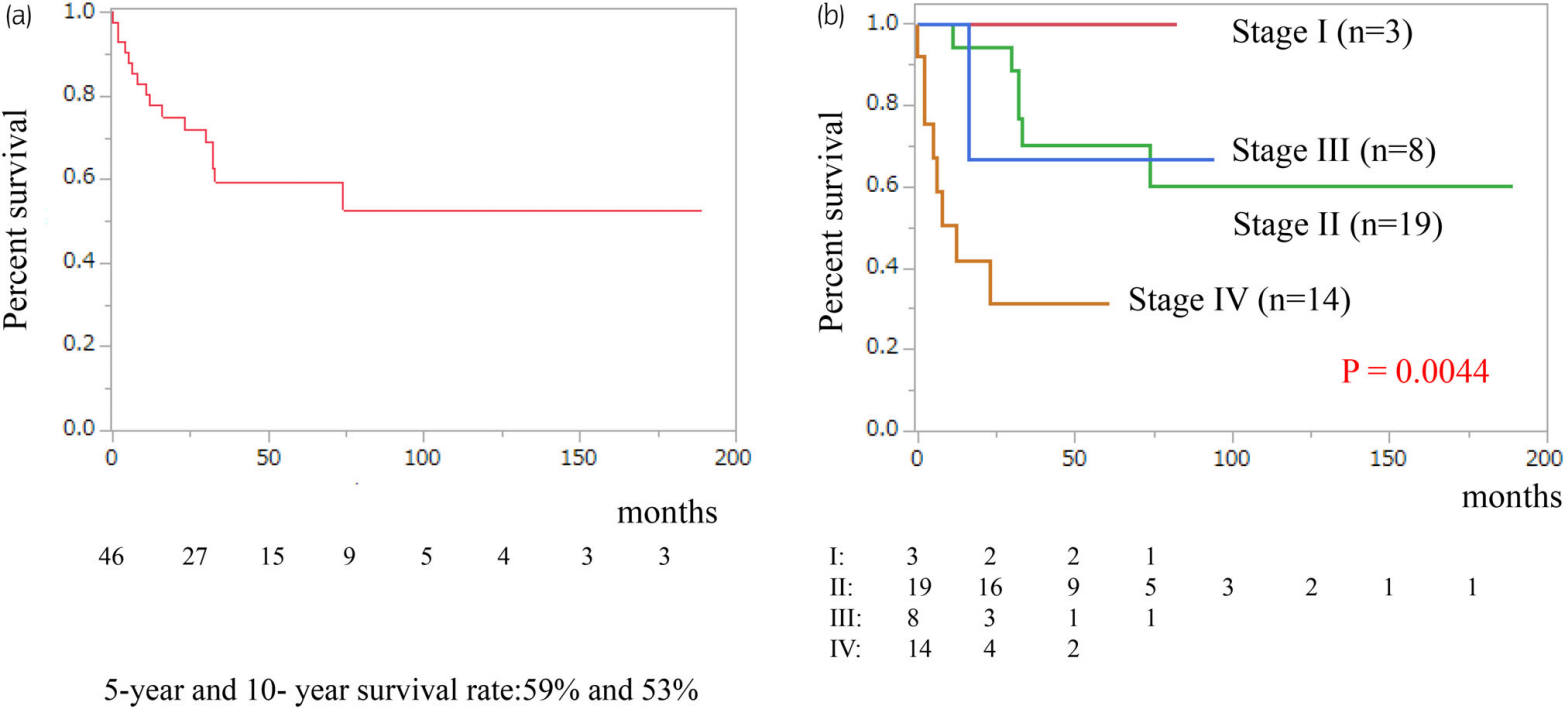
(a) The 5‐year and 10‐year survival rates of all patients were 59% and 53%, respectively. (b) The survival curves were shown by stage. The red line is stage I (*n* = 3), the green line is stage II (*n* = 7), the blue line is stage III (*n* = 19), and the orange line is stage IV (*n* = 14). The median overall survival of stage IV was 12 months.

**FIGURE 2:**
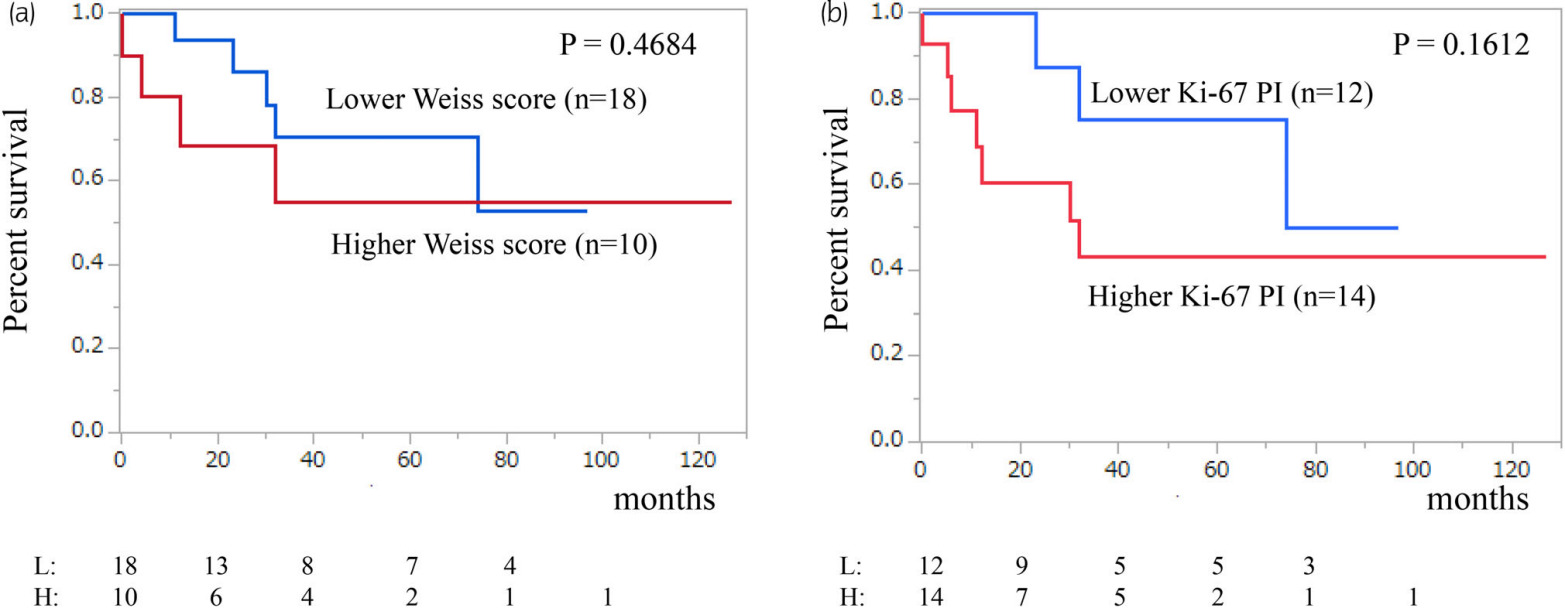
(a) Overall survival curves high (*n* = 10) and low (*n* = 18) of Weiss score. The blue line represents high score and the red line represents low score. (b) Overall survival curves high (*n* = 14) and low (*n* = 12) of Ki‐67 proliferation index. The blue line represents high score and the red line represents low score. No significant difference was observed between the two groups (*p* = 0.4684), (*p* = 0.1612).

**FIGURE 3:**
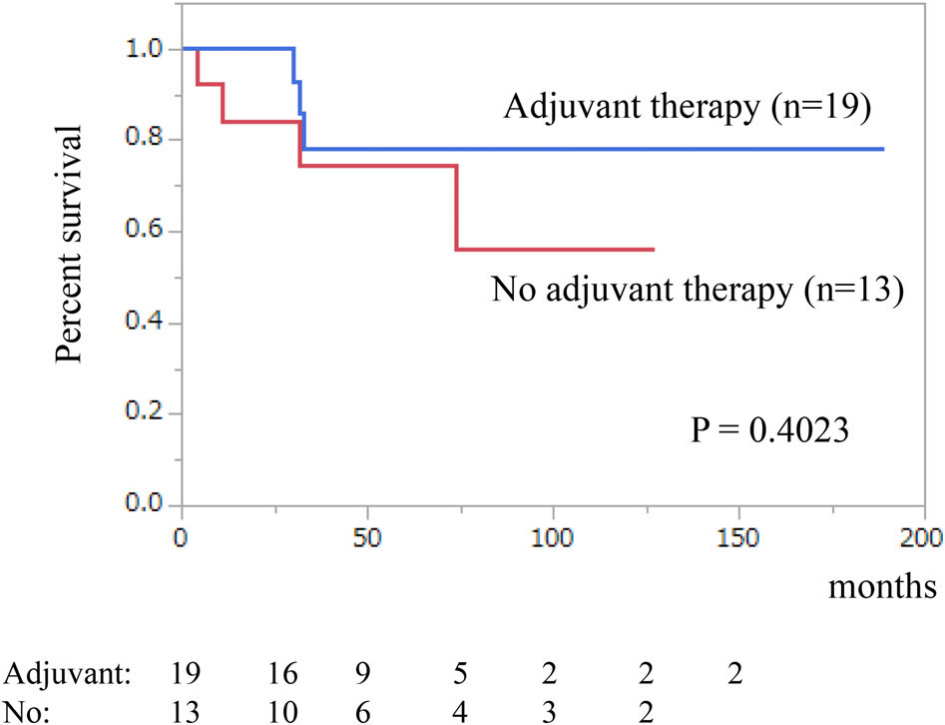
Overall survival curves were plotted for two groups: one with adjuvant treatment (*n* = 19), and another without adjuvant treatment (*n* = 13). The blue line represents the group with adjuvant treatment, while the red line represents the group without adjuvant treatment. The 5‐year survival rates were 78% and 75%, respectively, for these groups.

**FIGURE 4:**
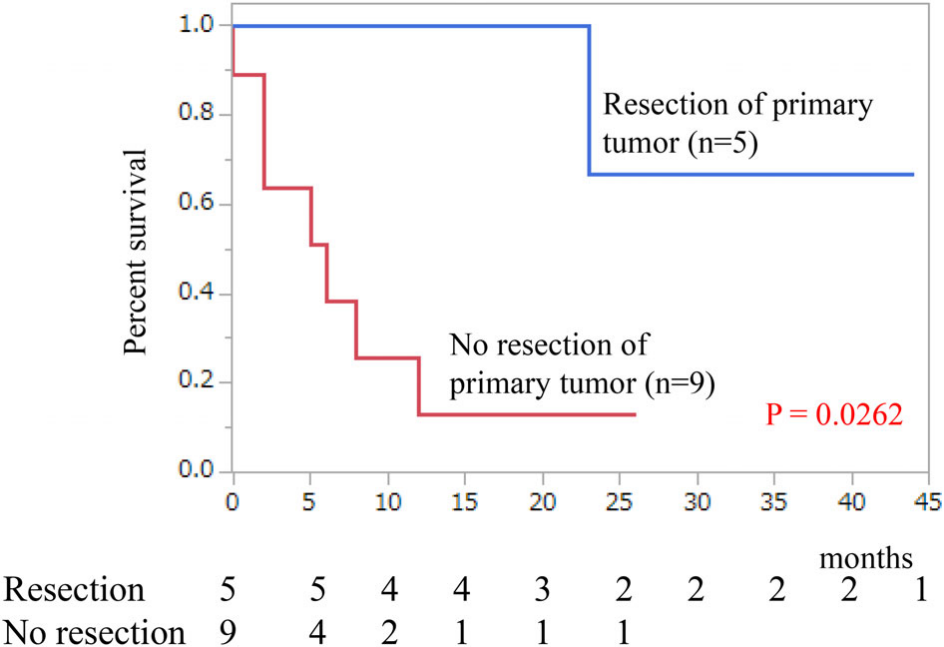
Survival curves depict two groups: one with primary tumor resection (*n* = 5) and the other without resection (*n* = 9) for stage IV ACC. The blue line represents patients who underwent resection, while the red line represents patients who did not undergo resection. The overall survival was significantly different between the two groups (*p* = 0.0262).

The median follow‐up time was 31 months, with corresponding five‐ and 10‐year overall survival rates of 59% and 53%, respectively (Figure 1a). Notably, overall survival differed significantly among TNM system for ACC of the American Joint Committee on Cancer/International Union Against Cancer stages (*p* = 0.0044; Figure 1b). Specifically, the median overall survival for patients with stage IV disease was 12 months. We classified cases with Weiss scores between 3 and 5 as lower and 6 and 9 as higher, but no significant difference in overall survival was observed between the two groups (*p* = 0.4684; Figure 2a). The value of Ki‐67 PI was divided into high and low groups at a cutoff value of 10%. However, no significant difference in overall survival was observed between the two groups (*p* = 0.1612; Figure 2b). Hormone secretion was not significantly associated with the prognosis (data not shown). Regarding the effects of treatments on the overall survival, adjuvant therapy had no significant impact on overall survival of postoperative patients at stages I–III (*p* = 0.4023; Figure 3). In contrast, primary tumor resection was significantly associated with prolonged overall survival in patients with stage IV disease (*p* = 0.0262; Figure 4). The ECOG PS of all these cases was 0 or 1; therefore, the ECOG PS did not differentiate between the presence or absence of primary tumor resection. Details of five cases with primary tumor resection were described below.

The first case involved a 73‐year‐old woman with liver metastasis who underwent primary tumor removal, followed by radiation for liver metastases. She died 23 months after diagnosis. The second patient was a 71‐year‐old woman who had pulmonary metastasis. The primary tumor was removed laparoscopically, and at the same time, the lung metastases were removed thoracoscopically. Remarkably, she survived for 44 months after diagnosis. The third case was a 43‐year‐old woman with liver metastasis. She underwent laparotomy to remove both the primary and the metastatic liver tumor. She survived for 6 months after diagnosis. The fourth case involved a 71‐year‐old man who had liver metastases, and the metastatic lesions were removed simultaneously with the primary tumor. He survived for 61 months after diagnosis. The fifth case involved a 73‐year‐old woman with bone metastasis, where only the primary tumor was removed. She survived for 18 months after diagnosis. In three of the five cases, the metastatic tumor was removed concurrently with the primary tumor. In addition, one patient underwent radiotherapy for metastatic lesions. These findings suggest a potential impact of localized treatment at metastatic lesions.

### Serum hormone level in ACC patients

**FIGURE 5:**
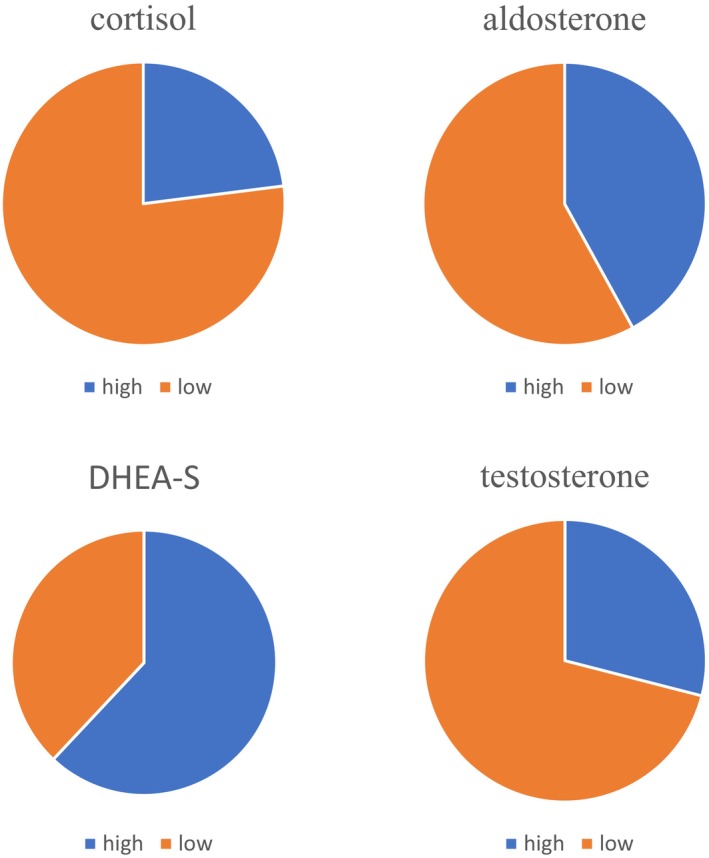
Incidence of hormone hypersecretion varied. The percentages of high plasma hormone levels in our cases were 23%, 42%, 62%, and 29% for cortisol, aldosterone, DHEA‐S, and testosterone, respectively.

Plasma hormone levels in patients with ACC are shown in Table 3 and Figure 5. Plasma cortisol, aldosterone, testosterone, and DHEA‐S levels were measured in 39, 33, 17, and 29 patients, respectively. Among the cases evaluated for plasma hormones, 23%, 42%, 29%, and 62% showed higher levels than the upper normal limits, respectively.

**TABLE 3:** Values of plasma hormone in the ACC patients.

	Number of patients	Median	Interquartile range	Normal range
ACTH (pg/mL)	36	7.2	(2.0, 18.8)	9–52
Testosterone (ng/mL)	17	3.2	(1.0, 10.7)	
Male	6	3.4	(1.9, 41.7)	2.0–7.5
Female	11	1.8	(0.8, 11.1)	0.1–0.5
Cortisol (μg/dL)	39	13.8	(10.1, 19)	2.7–15.5
Aldosterone (pg/mL)	33	122	(85.5, 266)	30–160
DHEA‐S (μg/dL)	29	267	(92.5, 1269)	
Male	11	267	(116, 1520)	
Female	18	281.5	(76, 1174.5)	

## DISCUSSION

Five‐year survival rates of ACC patients with stages I to II, III, and IV are 60% to 80%, 30% to 50%, and less than 25%, respectively.^4^, ^13^ In our study, the 5‐year survival rates according to the stage were 100%, 70%, 67%, and 31%, respectively. Notably, the prognosis of patients across all stages seemed to improve. Aggressive removal of the primary tumor, even at stage IV, and even in the event of recurrence, along with surgical treatment and radiation therapy, potentially contributed to these overall prognosis improvements. The patient who underwent metastasectomy six times was confirmed to be alive after a 35‐month observation period. In patients with metastatic disease, many institutions empirically select resection of the primary tumor as part of the treatment in the absence of efficient systemic therapies. Srougi et al. pooled 339 patients with metastatic ACC, with and without primary tumor extirpation. Their findings indicate a significantly favorable prognosis in the group that underwent primary tumor extirpation.^6^ In this study, the prognosis was significantly better in patients who underwent primary tumor resection, although the number of patients was small. Therefore, the decision regarding resection of the primary tumor in ACC patients with metastasis should be carefully considered on a case‐by‐case basis.

Surgical resection of the ACC is considered the primary treatment for localized diseases.^4^ Laparoscopic surgery is the standard treatment for benign adrenal tumors. However, no consensus regarding the optimal resection technique for ACC exists. Using a systematic review and meta‐analysis approach, Nakanishi et al. compared open *vs* laparoscopic surgery for ACC and reported that open surgery had a lower positive resection margin rate and better 3‐year overall and recurrence‐free survival rates.^14^ However, they concluded that laparoscopic surgery was more effective in selected cases because of a significantly shorter hospital stay.^14^ Delman et al. compared open *vs* laparoscopic surgery for ACC and reported that the overall survival was the same for both.^15^ Additionally, Cavallaro et al. recommended open surgery for tumors larger than 8 cm.^16^ In the laparoscopic surgery group of the present study, 29% of patients were classified as T1, 64% as T2, 7% as T3, and no T4 cases were observed, while in the open surgery group, 6% were classified as T1, 71% as T2, 6% as T3, and 18% as T4. Although no significant difference was observed in the ENSAT stages between the laparoscopic and open surgery groups, more T4 cases tended to be treated with open surgery. Consistent with previous reports, our results showed that laparoscopic surgery was performed in selected cases.

Adjuvant mitotane therapy is recommended for patients after complete surgical resection who have either stage III or stage IV disease or high‐grade disease of any stage (Ki‐67 PI >10%).^17^ Pathologists generally classify the malignant potential of ACC as high or low grade according to the mitotic count, whereas ENSAT treatment algorithms often rely on the Ki‐67 PI. Tumors with a Ki‐67 PI of 10% or less are considered low risk, whereas those with a PI of more than 10% are considered high risk.^18^, ^19^ For example, there is no evidence supporting adjuvant mitotane recommendation for patients with stage I–III and Ki‐67 PI <10% after R0 resection (ADIUVO trial, ClinicalTrials.gov identifier: 777244). There are currently ongoing clinical trials (ADIUVO‐2 trial, ClinicalTrials.gov identifier: NCT03583710) for adjuvant treatment of high‐risk cases (stage I–III and Ki‐67 PI >10%) of ACC with mitotane alone and mitotane plus cisplatin and etoposide, with the results expected around 2025.

EDP plus mitotane (EDP‐M) is currently the standard chemotherapy regimen in the first‐line treatment of advanced ACC.^20^ In a randomized phase‐III First International Randomized Trial in Locally Advanced and Metastatic (FIRM‐ACT) study, the overall survival of patients with advanced ACC treated with EDP‐M tended to be prolonged, but no significant difference was observed when compared with streptozotocin and mitotane (Sz‐M) therapy. Nevertheless, EDP‐M significantly prolonged the time of tumor progression compared with Sz‐M.^4^ Despite the advantage of EDP‐M, the mean survival rate of patients with advanced ACC is still frustrating. Currently, immunotherapy has revolutionized cancer treatment, exhibiting promising anti‐tumor activity in different solid tumors, including ACC. In a phase II study of pembrolizumab that enrolled 39 patients with advanced ACC, the clinical activity of 200 mg pembrolizumab every 3 weeks was assessed regardless of the prior therapy received. This study reported an objective response rate of 23%, a disease control rate of 52%, and a median overall survival of 24.9 months.^21^ Future clinical results are highly anticipated.

Functional tumors account for approximately 60% of all ACC cases.^1^ However, symptoms caused by hormone over‐secretion are evoked in around 40% of all cases of ACC. Due to the retrospective nature of this study, hormone measurement items and the number of cases measured varied and were small, making it difficult to comment on their relationship with symptoms or the amount of hormone secretion. Based on these results, we strongly recommend performing hormone measurements without fail when diagnosing an adrenal tumor.

Our study has several limitations. Firstly, it was a retrospective analysis of ACC patients treated across 10 university hospitals. Secondly, histological confirmation was not conducted in 7 (15%) of 46 cases. Thirdly, each hospital had a slightly different treatment strategy, which may have affected the prognostic outcomes. Nevertheless, considering that ACC is a rare cancer, the present report provides real‐world data on its clinicopathological characteristics.

In conclusion, the present study revealed that patients with metastatic ACC had the poorest prognosis, whereas resection of the primary tumor for metastatic ACC was significantly associated with prolonged overall survival. In contrast, adjuvant mitotane did not have a significant effect on overall survival. We anticipate that the findings of this study will contribute to future medical treatment of ACC in Japan.

## AUTHOR CONTRIBUTIONS

**Shotaro Nakanishi:** Conceptualization; Data curation; Formal analysis; Writing—original draft. **Yumi Fukushima:** Data curation; Resources; Writing—review & editing. **Junichi Inokuchi:** Data curation; Resources; Writing—review & editing. **Tomoaki Hakariya:** Data curation; Writing—review & editing; Resources. **Hiroaki Kakinoki:** Data curation; Resources; Writing—review & editing. **Hideki Enokida:** Data curation; Resources; Writing—review & editing. **Katsuaki Chikui:** Data curation; Resources; Writing—review & editing. **Hirofumi Matsuoka:** Data curation; Resources; Writing—review & editing. **Toshitaka Shin:** Data curation; Resources; Writing—review & editing. **Shoichiro Mukai:** Data curation; Resources; Writing—review & editing. **Tomomi Kamba:** Data curation; Resources; Writing—review & editing. **Masatoshi Eto:** Data curation; Resources; Writing—review & editing. **Ryoichi Imamura:** Data curation; Resources; Writing—review & editing. **Mitsuru Noguchi:** Data curation; Resources; Writing—review & editing. **Tsukasa Igawa:** Data curation; Resources; Writing—review & editing. **Nobuhiro Haga:** Data curation; Resources; Writing—review & editing. **Toshiyuki Kamoto:** Data curation; Resources; Writing—review & editing. **Naohiro Fujimoto:** Data curation; Resources; Writing—review & editing. **Seiichi Saito:** Conceptualization; Writing—review & editing; Supervision.

## CONFLICT OF INTEREST STATEMENT

The authors declare no conflict of interest.

## APPROVAL OF THE RESEARCH PROTOCOL BY AN INSTITUTIONAL REVIEW BOARD

This study was approved by the Ethics Committee of the University of the Ryukyus and each university hospital (Approval No. 1442).

## INFORMED CONSENT

N/A.

## REGISTRY AND THE REGISTRATION NO. OF THE STUDY/TRIAL

N/A.

## ANIMAL STUDIES

N/A.
